# Application of the European Kidney Function Consortium Equation to Estimate Glomerular Filtration Rate: A Comparison Study of the CKiD and CKD-EPI Equations Using the Korea National Health and Nutrition Examination Survey (KNHANES 2008–2021)

**DOI:** 10.3390/medicina60040612

**Published:** 2024-04-08

**Authors:** Seungho Lee, Gun-Hyuk Lee, Hanah Kim, Hyun Suk Yang, Mina Hur

**Affiliations:** 1 Department of Preventive Medicine, College of Medicine, Dong-A University, Busan 49201, Republic of Korea; lgydr1@gmail.com; 2 Environmental Health Center for Busan, Dong-A University, Busan 49201, Republic of Korea; 3 Department of Laboratory Medicine, Konkuk University School of Medicine, Seoul 05030, Republic of Korea; leegunhyuk93@gmail.com (G.-H.L.); md.hkim@gmail.com (H.K.); 4 Department of Cardiovascular Medicine, Research Institute of Medical Science, Konkuk University School of Medicine, Seoul 05030, Republic of Korea

**Keywords:** glomerular filtration rate, equation, EKFC, CKD-EPI, comparison, agreement

## Abstract

*Background and Objectives*: The European Kidney Function Consortium (EKFC) equation has been newly proposed for estimating glomerular filtration rate (eGFR) across the spectrum of age. We compared the EKFC equation with the Chronic Kidney Disease Epidemiology Collaboration (CKD-EPI) equations in a large-scale Korean population. *Materials and Methods*: Using the representative Korean health examination data, the Korea National Health and Nutrition Examination Survey (KNHANES 2008–2021), the records of 91,928 subjects (including 9917 children) were analyzed. We compared the EKFC equation with CKiD, CKD-EPI 2009, and CKD-EPI 2021 equations and investigated their agreement across GFR categories. *Results*: In the total population, the CKD-EPI 2021 equation yielded the highest eGFR value, followed by the CKD-EPI 2009 and EKFC equations. In children, the distribution of eGFR differed significantly between the EKFC and CKiD equations (*p* < 0.001), with a wider range of eGFR values found with the CKiD equation. Each equation showed weak or moderate agreement on the frequency of the GFR category (κ = 0.54 between EKFC and CKD-EPI 2021; κ = 0.77 between EKFC and CKD-EPI 2009). The eGFR values found by the EKFC equation showed high or very high correlations with those by the CKiD, CKD-EPI 2009, and CKD-EPI 2021 equations (r = 0.85, 0.97, and 0.97, respectively). As eGFR values increased, bigger differences were observed between equations. *Conclusions*: This large-scale study demonstrates that the EKFC equation would be applicable across the entire age spectrum in Asian populations. It also underscores that national kidney health would be highly affected by an eGFR equation being implemented. Additional investigation and more caution would be warranted for the transition of eGFR equations.

## 1. Introduction

Although serum creatinine (sCr) is the most commonly used kidney biomarker, the clinical interpretation of sCr concentration is not straightforward due to its inherent vulnerability to muscle mass and consequent population characteristics such as age, sex, race, and nutritional habits, etc. [1]. Glomerular filtration rate (GFR) is a useful index for assessing kidney function. Since direct measurement of GFR using inulin clearance or radio-isotope is time-consuming and impractical in clinical practice, several sCr-based equations have been developed to estimate GFR [2]. Currently, the most widely used estimated GFR (eGFR) equations are the Chronic Kidney Disease Epidemiology Collaboration (CKD-EPI) equations for adults and the Chronic Kidney Disease in Children (CKiD) equation for children [2,3,4,5]. These equations, however, are known to have several limitations, especially in the age group transitioning from adolescents to adults [6,7].

Recently announced CKD-EPI 2021 equations were refit without a race coefficient, and the task force created by the National Kidney Foundation (NKF) and the American Society of Nephrology (ASN) recommended immediately replacing older sCr-based eGFR equations with the new CKD-EPI 2021 equation [8,9]. It has been reported that this new race-free equation would underestimate measured GFR (mGFR) in Black people and overestimate it in non-Black people [10]. Such a change was expected to increase the prevalence of chronic kidney disease (CKD) among Black people, and yield similar or lower CKD prevalence among non-Black people [10,11,12].

Contrary to the NKF-ASN task force recommendation, the European Federation of Clinical Chemistry and Laboratory Medicine (EFLM) recommended not to implement the CKD-EPI 2021 equation in European laboratories, and rather supported implementing the newly suggested European Kidney Function Consortium (EKFC) equation [13,14]. The EKFC equation can be used across the full age range, without any discontinuity at the transition between pediatric and adult nephrology care [13]. It has been shown that the EKFC equation also performed well in the populations other than white Europeans, notably in Black populations of Europe, Brazil, and Africa [15,16,17].

Even though these newly proposed eGFR equations can be applied globally, their evaluation is mandatory before being adopted in regions other than North America or Europe, and is important, considering the conflicting recommendations on the new equations. Several studies have explored the performance of these new equations in Asian populations [18,19,20]; however, there has been only one large-scale study, and there has been no study conducted in the pediatric age group. Thus, it would be necessary to compare and verify the results of each equation in Asian populations including children, adolescent, and adults. In this study, we compared different equations (CKiD, CKD-EPI 2009, CKD-EPI 2021, and EKFC) and investigated the agreement across the GFR categories using the representative Korean health examination data.

## 2. Materials and Methods

### 2.1. Study Population

The Korea National Health and Nutrition Examination Survey (KNHANES) is a nationwide cross-sectional survey that has been conducted by the Korea Centers for Disease Control and Prevention (KCDC) since 1998 [21]. It has been performed periodically to assess the health and nutritional status in the general Korean population and consists of three component surveys: health interview, health examination, and nutrition survey. The health interview and health examination were conducted by trained staff members, including physicians. Participants were selected using proportional-allocation systematic sampling with multi-stage stratification, and their information on socioeconomic status, health-related behaviors, quality of life, healthcare utilization, anthropometric measures, biochemical and clinical profiles for non-communicable diseases, and dietary intakes was collected. The KNHANES provides statistics for health-related policies and the research infrastructure for studies on risk factors and diseases in Korea [22]. The KCDC publishes the Korea Health Statistics each year, and microdata are publicly available through the KNHANES website (http://knhanes.cdc.go.kr, accessed on 1 February 2024).

For the present study, we used the data from the KNHANES 2008–2021 (N = 115,587). Among the 115,587 eligible data, 23,659 participants with missing data were excluded, and the remaining 91,928 participants (41,113 males and 50,815 females) were included in the final dataset. Written informed consent was obtained from all participants or their parents (in the case of pediatric participants), and the study protocol was approved by the Institutional Review Board of the KCDC (IRB number: 2008-04EXP-01-C, 2009-01CON-03-2C, 2010-02CON-21-C, 2011-02CON-06-C, 2012-01EXP-01-2C, 2013-07CON-03-4C, 2013-12EXP-03-5C, 2018-01-03-P-A, 2018-01-03-C-A, 2018-01-03-2C-A, 2018-01-03-3C-A).

### 2.2. Estimation of GFR and Categories

After at least eight hours of fasting, blood samples were collected in the morning and were analyzed at a central laboratory (Neodin Medical Institute, Seoul, Republic of Korea). sCr concentration was determined by the Jaffe rate-blanked and compensated method using a Hitachi automated analyzer 7600 (Hitachi, Tokyo, Japan) before 2013, and a Hitachi automated analyzer 7600-210 (Hitachi, Tokyo, Japan) until 2018. From 2019, sCr concentration was measured by Kinetic colorimetric assay using Cobas 8000 analyzer (Roche Diagnostics, Mannheim, Germany). Based on the sCr concentration, eGFR by CKiD equation was derived for children (≤18 years), eGFR by CKD-EPI equations were derived for adults (≥19 years), and eGFR by EKFC equation was derived for all age groups. The eGFR (mL/min/1.73 m^2^) was calculated using the four equations as follows.

(a) CKiD:

eGFR = 41.3 × (height/100)/(sCr/88.4).

height is expressed in centimeter in the equation.

(b) CKD-EPI 2009 equation:

eGFR = 141 × min (sCr/κ, 1)^α^ × max (sCr/κ, 1)^−1.209^ × 0.993^Age^ (× 1.018, if female).

κ is 0.9 (for males) and 0.7 (for females). α is −0.411 (for males) and −0.329 (for females). min and max indicate the minimum and the maximum of sCr/κ or 1, respectively.

(c) CKD-EPI 2021 equation:

eGFR = 142 × min (sCr/κ, 1)^α^ × max (sCr/κ, 1)^−1.200^ × 0.9938^Age^ (× 1.012, if female).

κ is 0.9 (for males) and 0.7 (for females). α is −0.302 (for males) and −0.241 (for females). min and max indicate the minimum and the maximum of sCr/κ or 1, respectively.

(d) EKFC equation:

eGFR = 107.3 × (sCr/Q)^−0.322^ (for aged 2–40 years & sCr/Q < 1); eGFR = 107.3 × (sCr/Q)^−1.132^ (for aged 2–40 years & sCr/Q ≥ 1); eGFR = 107.3 × (sCr/Q)^−0.322^ × 0.990^(Age − 40)^ (for aged over 40 years & sCr/Q < 1); eGFR = 107.3 × (sCr/Q)^−1.132^ × 0.990^(Age − 40)^ (for aged over 40 years & sCr/Q ≥ 1).

ln(Q) = 3.200 + 0.259 × age − 0.543 × ln(age) − 0.00763 × age^2^ + 0.0000790 × age^3^, for aged 2–25 years and males; ln(Q) = 3.080 + 0.177 × age − 0.223 × ln(age) − 0.00596 × age^2^ + 0.0000686 × A^3^, for aged 2–25 years and females; Q = 80 µmol/L, for aged over 25 years and males; Q = 62 µmol/L, for aged over 25 years and females.

According to the Kidney Disease Improving Global Outcomes (KDIGO) guidelines, six GFR categories were used to assess each eGFR (mL/min/1.73 m^2^, G1: ≥90, G2: 60–89, G3a: 45–59, G3b: 30–44, G4: 15–29, G5: <15). Reduced GFR corresponded to eGFR ≤ 60 mL/min/1.73 m^2^ [3].

### 2.3. Statistical Analysis

Data were expressed as median and interquartile range (IQR) for continuous variables and as numbers and percentages for categorical or binary variables. All continuous variables were tested for normality using Kolmogorov–Smirnov nonparametric tests. The full distribution of eGFR was displayed using violin plots with summary statistics, and the data probability density at different values was compared using survey strata by sex, age group (≤18, 19–25, 26–40, 41–60, and >60 years), and body mass index (BMI: kg/m^2^; <18.5, 18.5 to <23.0, 23.0 to <25.0, 25.0 to <30.0, and ≥30.0). The agreement on frequency of the KDIGO categories according to each eGFR equation was assessed using Cohen’s kappa (κ) with 95% confidence interval (CI), which was interpreted as follows: ≤0.20, none; 0.21–0.39, minimal; 0.40–0.59, weak; 0.60–0.79, moderate; 0.80–0.90, strong; and >0.90, nearly perfect [23]. The eGFRs by different equations were compared using the Passing–Bablok regression and Bland–Altman plots, according to the CLSI guidelines (EP09-ED3) [24]. The scatter plots were used to identify the correlation or difference between equations. In the Passing–Bablok regression, the correlation coefficients (r) were interpreted as follows: <0.30, negligible; 0.30–0.49, low; 0.50–0.69, moderate; 0.70–0.89, high; and ≥0.90, very high correlations [25]. In the Bland–Altman plots, the results were interpreted informally to observe how big the mean difference is and whether there is a trend of difference [26]. Using the receiver operating characteristic (ROC) curve analyses, the area under the curve (AUC) of each variable (eGFR equations, sCr, age, and BMI) was compared for predicting >95% difference between the two eGFR equations. The Mann–Whitney U test and Chi-squared test were used to compare variables or proportions, as appropriate. Statistical analyses were conducted using the MedCalc Statistical Software (version 22.019, MedCalc Software Ltd., Ostend, Belgium), and a two-tailed *p* value < 0.05 was considered statistically significant.

## 3. Results

**Figure 1:**
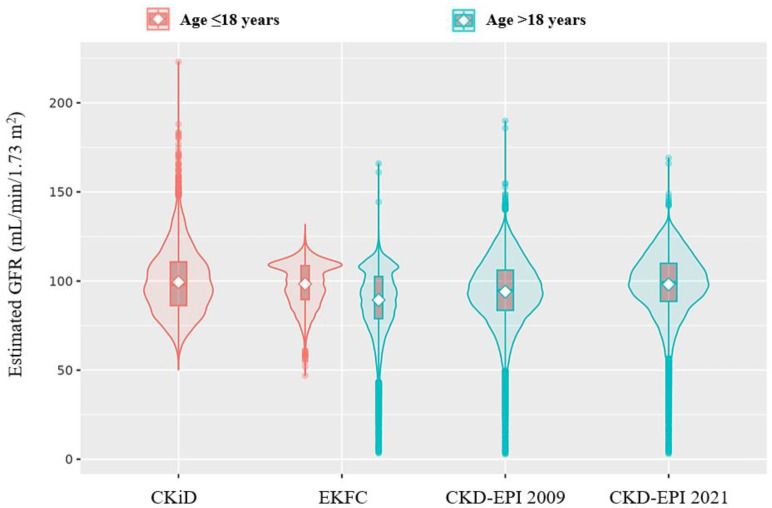
Distribution of estimated GFR with CKiD, EKFC, CKD-EPI 2009, and CKD-EPI 2021 equations.

Table 1 shows the distribution of clinical and laboratory parameters by age group. The median values of sCr concentration ranged from 0.68 to 0.81 mg/dL in the study population; sCr concentration showed higher values in males than in females across each age group (0.74–0.95 mg/dL vs. 0.62–0.71 mg/dL), with the lowest values found in the age group ≤18 years. Figure 1 shows the distribution of eGFR using each equation. In the age group >18 years, the CKD-EPI 2021 equation showed the highest value, followed by the CKD-EPI 2009 and EKFC equations (median [IQR]; 99.29 [88.56–109.94] vs. 94.68 [83.55–106.13] vs. 90.69 [78.88–102.55] mL/min/1.73 m^2^, respectively). In the age group ≤18 years, the median (IQR) eGFR values using the CKiD and EKFC equations were 98.00 (86.21–110.74) and 100.09 (89.61–108.67) mL/min/1.73 m^2^, respectively. Although the difference of median eGFR values was 2.09 mL/min/1.73 m^2^, the distribution of eGFR values showed a more than two-fold wider range when using the CKiD equation than using the EKFC equation (range, 172.74 vs. 84.81 mL/min/1.73 m^2^). Differently from the EKFC equation, the CKiD equation showed the long-tailed outside points above the upper adjacent value.

Table 2 shows the comparison of eGFR using each equation. In the total population, the CKD-EPI 2021 equation yielded the highest eGFR value, followed by the CKD-EPI 2009 and EKFC equations. Such findings were constantly observed when they were stratified into subgroups of sex, age, and BMI. Generally, the eGFR values tended to decrease with increasing age among adults; however, eGFR values found by the EKFC equation increased slightly in the young adult population (age: 19–40 years). As BMI increased, the eGFR value decreased except for in the severely obese group (BMI > 30 kg/m^2^). The frequency of KDIGO categories according to each eGFR equation is presented in Table 3. In the adult population, the combined proportion of G1 and G2 was 96.2% by the CKD-EPI 2009, 97.4% by the CKD-EPI 2021, and 94.7% by the EKFC equation, showing significant differences (all *p* < 0.0001). Each equation showed weak or moderate agreement on the frequency of KDIGO categories (κ = 0.54 between the EKFC and CKD-EPI 2021; κ = 0.77 between the EKFC and CKD-EPI 2009). In children, the combined proportion of G1 and G2 was same (99.8%) by both the CKiD and EKFC equations, although each G1 and G2 proportion showed significant differences between the CKiD and EKFC equations (all *p* < 0.0001).

**Figure 2:**
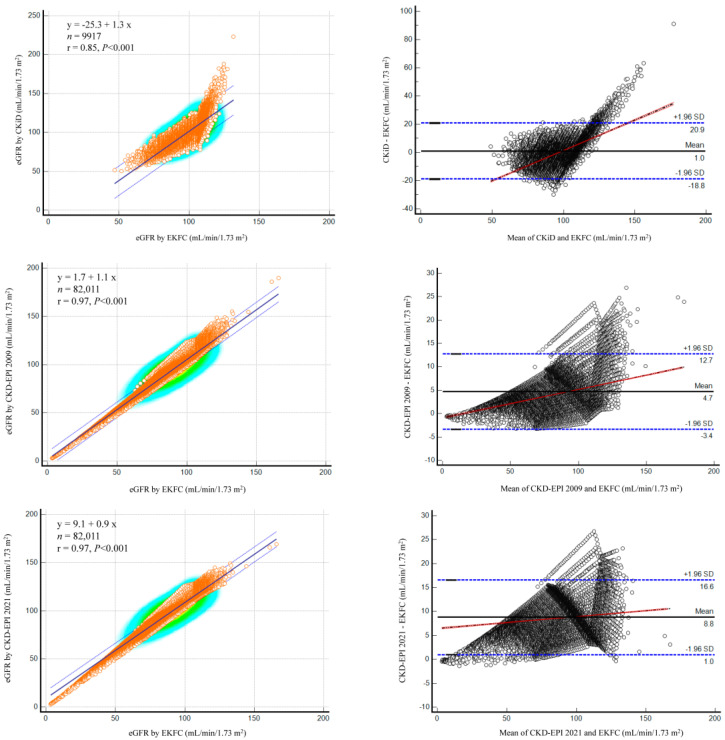
Relationships between CKiD, CKD-EPI 2009, and CKD-EPI 2021 with EKFC equations.

**Figure 3:**
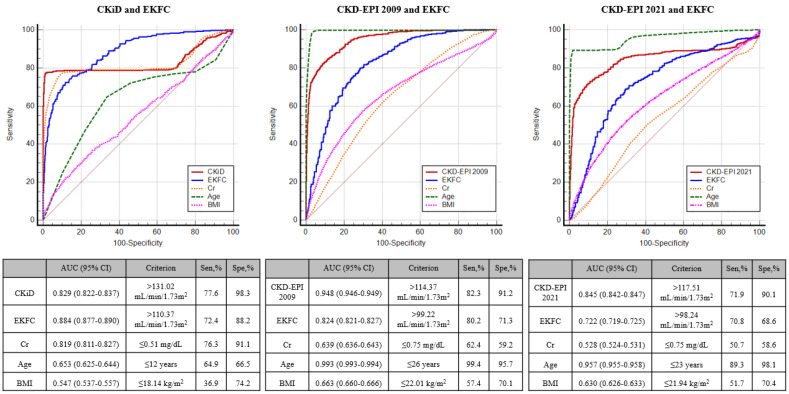
Receiver operating characteristic (ROC) curves for predicting >95% difference between the two eGFR equations. Abbreviations: AUC, are under the curve; Sen, sensitivity; Spe, specificity.

Figure 2 shows the relationship between each equation; eGFR values found by the CKiD, CKD-EPI 2009, and CKD-EPI 2021 equations showed high or very high correlations with those by the EKFC equation (r = 0.85, 0.97, and 0.97, respectively). However, the existence of proportional bias indicated that the two equations do not agree equally through the range of eGFR values. As eGFR values increased, bigger differences were observed. When we further searched for the factors predicting >95% difference between the two eGFR equations, age was the strongest predictor in adults (AUC = 0.993 with the cut-off of ≤26 years between CKD-EPI 2009 and EKFC equations) (Figure 3). In children, the EKC equation with an eGFR value > 110.37 mL/min/1.73 m^2^ was the strongest predictor for such a difference (AUC = 0.884), with the proportion of 14.8% (1465/9917 subjects). Compared with the low EKFC subgroup, the high EKFC subgroup showed significant differences in terms of demographic, clinical, and laboratory data (all *p* < 0.001 except for HbA1c) (Table 4). The proportion with >95% difference of eGFR values between the CKiD and EKFC equations was significantly higher in the high EKFC group than in the low EKFC group (22.9% vs. 1.5%, *p* < 0.001).

## 4. Discussion

This was a large-scale study that was conducted in the Asian population spanning children, adolescents, and adults to explore the application of newly proposed eGFR equations. Using a representative Korean health data that was collected for 14 years (the KNHANES data 2008–2021), we analyzed 91,928 nationwide records including 9917 for children. Our data showed that there was a consistent difference in eGFRs by each equation across all age groups. In adults, compared with the CKD-EPI 2009 equation, the EKFC and CKD-EPI 2021 equations shifted the distribution of eGFRs significantly in the opposite direction (*p* < 0.001). Our finding is in line with previous studies [15,16,17,18,19]. A previous large-scale study on the Korean general population (approximately 100,000 adults) showed potential implications for CKD prevalence across different eGFR equations [18]. Given that the current CKD prevalence in the adult population (5.8% as of 2022) is derived from the CKD-EPI 2009 equation in Korea, implementing these new eGFR equations would bring about a sizable change in the CKD prevalence; it would be overestimated by the EKFC equation, while it would be underestimated by the CKD-EPI 2021 equation [18].

In the present study, one of the noticeable findings is the distribution of eGFRs in children, showing a significant difference between the CKiD and EKFC equations (*p* < 0.001). The EKFC equation showed a relatively concentrated distribution of eGFR; on the contrary, the CKiD equation showed a much wider range of eGFR values, especially with an upward long tail. Regarding the frequency of KDIGO categories, the two equations showed moderate agreement. However, the EKFC equation showed a significantly higher G1 proportion than the CKiD equation (73.8% vs. 67.5%, *p* < 0.0001), and the same proportional gap of 6.3% was observed in the G2 proportion by each equation (26.0% vs. 32.3%, *p* < 0.0001). Accordingly, the combined proportion (G1 and G2) implying normal kidney function accounted for 99.8% of the subjects by using both equations. Given that our data were derived from the general pediatric population, most of whom are assumed to have normal kidney function, the EKFC equation seems to be a reasonable clinical tool for assessing GFR in the pediatric population. Further studies are warranted to validate the applicability of the EKFC equation in pediatric populations with various ethnicities and clinical settings.

Although the equations showed high or very high correlations in the eGFR values, they also showed a proportional difference as eGFR values increased. Of note, age was the strongest predictor of eGFR difference in adults between the CKD-EPI 2009 and EKFC equations (AUC = 0.993) with the age cut-off of ≤26 years. Our data demonstrate that eGFR may differ considerably in the young age group (18–25 years); in addition to the age cut-off of 40 years, it also provides the rationale of using the proposed cut-off of 26 years for calculating Q value in the EKFC equation [6,13,17,27]. At the transition age of 18 years, implausible increases in eGFR have been observed when switching from the height-dependent CKiD equation in children to the age-dependent CKD-EPI equation in adults [3,7]. Lack of height data would also preclude an automatic reporting of CKiD-based eGFR in clinical laboratories [7]. In our data, approximately 15% of the pediatric population (1465/9917 subjects) showed the eGFR > 110.37 mL/min/1.73 m^2^ by the EKFC equation. In this high EKFC group, median eGFR values between the CKiD and EKFC equations showed a significant difference than in the low EKFC group (12.1 vs. −0.9 mL/min/1.73 m^2^); it implies that as GFR increases, eGFRs found by the CKiD equation may increase more profoundly than eGFRs found by the EKFC equation (median eGFR difference: 30.5 vs. 16.2 mL/min/1.73 m^2^, respectively). This finding also suggests that flawed increases in kidney function would be decreased when using EKFC equation, although all current eGFR equations have their own limits of accuracy and precision [17].

This study is limited in that it was a retrospective analysis using cross-sectional data from the national health and nutrition examination survey. During the 14-year period, sCr concentration was measured with three different methods, and this approach may have the potential to bias the results. Additionally, due to the limited data availability, we could neither analyze eGFR in comparison with mGFR nor extend our evaluation including cystatin-C-based eGFR equations [17,28,29]. Moreover, without in-depth clinical information and repeated sCr measurements, we could not explore the association between eGFR changes and detailed clinical outcomes in various clinical settings [30,31,32,33,34]. Nevertheless, the strength of our study is that the present data were obtained from a large number of enrolled subjects, who reflect the general Korean population.

## 5. Conclusions

In conclusion, considering the rarity of this kind of large-scale study in Asian populations, especially in pediatric populations worldwide, our study provides some fundamental data that can form the basis of further research. Our data demonstrate that different eGFR equations would affect the epidemiological data on kidney function and lead to considerable changes in national kidney health. The current usage of eGFR equations is not uniform across different healthcare systems, and the transition of eGFR equations to the newly proposed equations is expected to occur gradually in a heterogenous way. Considering the conflicting recommendations on the newly proposed equation, more caution would be mandatory regarding the transition to eGFR equations in each region. For not only national kidney health, but also global kidney health, an increasing body of evidence should be accumulated to reach the international consensus on a unified appropriate eGFR equation. Although the limits of each eGFR equation should be acknowledged, we can conclude that the EKFC equation would be applicable across the full age spectrum, including children and young adults, in the Asian populations.

## Figures and Tables

**Table 1:** Distribution of clinical and laboratory parameters by age group.

	Age Group				
	≤18 Years	19–25 Years	26–40 Years	41–60 Years	>60 Years
Total					
N	9917	6225	19,309	30,674	25,803
BMI (kg/m^2^)	20.2 (18.0–22.9)	21.7 (19.7–24.4)	23.0 (20.7–25.7)	23.9 (21.9–26.0)	24.0 (22.0–26.0)
SBP (mm Hg)	106 (100–113)	108 (101–116)	109 (102–118)	117 (107–128)	127 (116–139)
DBP (mm Hg)	66 (60–72)	71 (65–76)	73 (68–80)	78 (71–85)	74 (68–81)
sCr (mg/dL)	0.68 (0.59–0.80)	0.80 (0.69–0.93)	0.80 (0.68–0.93)	0.79 (0.68–0.92)	0.81 (0.70–0.96)
Glucose (mg/dL)	90 (86–95)	88 (84–92)	91 (86–97)	96 (89–104)	99 (92–112)
Hb A1c (%)	5.4 (5.2–5.6)	5.3 (5.1–5.5)	5.4 (5.2–5.6)	5.6 (5.4–5.9)	5.9 (5.6–6.4)
TC (mg/dL)	159 (142–177)	170 (151–190)	184 (163–207)	195 (172–220)	186 (160–212)
TG (mg/dL)	74 (53–104)	75 (56–108)	94 (64–147)	114 (77–173)	117 (83–165)
Male					
N	5298	2841	8448	13,247	11,279
BMI (kg/m^2^)	20.5 (18.2–23.5)	23.0 (20.8–25.6)	24.5 (22.4–26.9)	24.5 (22.6–26.4)	23.7 (21.7–25.6)
SBP (mm Hg)	109 (102–116)	114 (107–121)	115 (108–123)	120 (111–130)	126 (115–137)
DBP (mm Hg)	67 (60–73)	73 (68–80)	78 (72–85)	81 (74–88)	74 (68–81)
sCr (mg/dL)	0.74 (0.60–0.88)	0.94 (0.88–1.02)	0.95 (0.88–1.03)	0.94 (0.86–1.02)	0.95 (0.85–1.08)
Glucose (mg/dL)	91 (87–96)	89 (84–93)	93 (88–99)	99 (92–109)	101 (93–114)
Hb A1c (%)	5.4 (5.3–5.6)	5.3 (5.1–5.5)	5.5 (5.3–5.7)	5.7 (5.4–6.0)	5.9 (5.5–6.4)
TC (mg/dL)	155 (138–173)	169 (150–190)	191 (168–214)	194 (171–218)	178 (154–203)
TG (mg/dL)	72 (51–102)	89 (63–129)	125 (85–190)	141 (96–213)	115 (81–167)
Female					
N	4619	3384	10,861	17,427	14,524
BMI (kg/m^2^)	19.8 (17.9–22.1)	20.8 (19.1–23.0)	21.8 (19.9–24.2)	23.3 (21.4–25.7)	24.2 (22.2–26.4)
SBP (mm Hg)	104 (98–110)	104 (98–110)	105 (99–111)	114 (105–126)	128 (117–140)
DBP (mm Hg)	66 (60–71)	69 (63–74)	70 (65–76)	75 (69–82)	74 (68–81)
sCr (mg/dL)	0.62 (0.55–0.70)	0.70 (0.63–0.76)	0.70 (0.62–0.76)	0.70 (0.63–0.77)	0.71 (0.64–0.81)
Glucose (mg/dL)	90 (85–94)	87 (83–91)	89 (85–95)	94 (88–101)	98 (91–110)
Hb A1c (%)	5.4 (5.2–5.6)	5.3 (5.1–5.5)	5.40 (5.2–5.6)	5.6 (5.4–5.9)	5.9 (5.6–6.3)
TC (mg/dL)	162 (147–180)	171 (153–190)	179 (159–201)	196 (174–221)	192 (166–219)
TG (mg/dL)	77 (56–105)	68 (51–92)	75 (55–111)	97 (69–143)	118 (85–164)

Data are presented as median (interquartile range). Male (10–80 years), female (9–80 years). Abbreviations: N, number; BMI, body mass index; SBP, systolic blood pressure; DBP, diastolic blood pressure; sCr, serum creatinine; TC, total cholesterol; TG, triglyceride; Hb, hemoglobin.

**Table 2:** Comparison of estimated glomerular filtration rates (mL/min/1.73 m^2^) using each equation.

	CKD-EPI 2009		CKD-EPI 2021		EKFC	
Category	N	Median (IQR)	N	Median (IQR)	N	Median (IQR)
Total	82,011	94.68 (83.55–106.13)	82,011	99.29 (88.56–109.94)	91,928	91.84 (79.90–103.95)
Sex						
Male	35,815	92.03 (81.24–102.87)	35,815	96.91 (86.26–107.05)	41,113	89.86 (78.99–100.95)
Female	46,196	97.02 (85.61–108.71)	46,196	101.30 (90.53–112.05)	50,815	93.17 (80.91–105.82)
Age (years)						
≤18	9917 *	98.00 (86.21–110.74)		-	9917	100.09 (89.61–108.64)
19–25	6225	119.38 (106.36–125.36)	6225	122.31 (109.13–127.45)	6225	104.92 (94.31–109.67)
26–40	19,309	109.40 (97.57–115.83)	19,309	113.27 (101.23–118.75)	19,309	107.37 (95.83–110.33)
41–60	30,674	96.82 (86.64–103.19)	30,674	101.70 (91.12–107.34)	30,674	93.38 (85.17–100.46)
>60	25,803	82.47 (70.91–89.96)	25,803	88.30 (76.04–95.32)	25,803	75.53 (65.69–82.77)
BMI (kg/m^2^)						
<18.5	3792	103.23 (88.82–115.62)	3792	106.79 (93.47–118.51)	6804	100.28 (88.24–108.71)
18.5 to <23.0	31,672	97.40 (86.02–108.98)	31,672	101.63 (91.02–112.51)	36,211	93.78 (81.95–105.82)
23.0 to <25.0	19,097	92.61 (82.10–102.94)	19,097	97.33 (87.16–107.03)	20,212	88.91 (77.96–100.07)
25.0 to <30.0	23,560	91.95 (80.65–102.46)	23,560	96.85 (85.55–106.62)	24,581	88.31 (77.06–99.84)
≥30.0	3890	97.45 (84.73–109.17)	3890	101.81 (89.65–112.72)	4120	94.16 (81.27–106.29)

* The eGFR was derived using the CKiD equation for this age group (≤18 years). Abbreviations: N, number; IQR, interquartile range; BMI, body mass index.

**Table 3:** Frequency of KDIGO categories according to each eGFR equation.

	G1: ≥90	G2: 60 to <90	G3a: 45 to <60	G3b: 30 to <45	G4: 15 to <30	G5: <15	Kappa * (95% CI)
	N (%)	N (%)	N (%)	N (%)	N (%)	N (%)	
Children (N = 9917)							
CKiD	6692 (67.5)	3206 (32.3)	19 (0.2)	-			0.64 (0.62–0.65)
EKFC	7323 (73.8)	2579 (26.0)	15 (0.2)	-			
Adults (N = 82,011)							
CKD-EPI 2009	50,255 (61.3)	28,590 (34.9)	2371 (2.9)	577 (0.7)	158 (0.2)	60 (0.1)	0.77 (0.77–0.77)
CKD-EPI 2021	59,195 (72.2)	20,637 (25.2)	1600 (2.0)	400 (0.5)	126 (0.2)	53 (0.1)	0.54 (0.53–0.54)
EKFC	42,219 (51.5)	35,427 (43.2)	3343 (4.1)	794 (1.0)	172 (0.2)	56 (0.1)	
Total (N = 91,928)							
EKFC	49,542 (53.9)	38,006 (41.3)	3358 (3.7)	794 (0.9)	172 (0.2)	56 (0.1)	

* Each kappa value represents the agreement with the eGFR derived from EKFC equation. Abbreviations: N, number; CI, confidence interval.

**Table 4:** Comparison between groups with high EKFC (>110.37 mL/min/1.73 m^2^) and low EKFC in subjects aged 18 or younger.

	High EKFC (N = 1465)	Low EKFC (N = 8452)	*p* Value
Female, N	859 (58.6%)	3760 (44.5%)	<0.001
Age, years	13.0 (11.0–15.0)	14.0 (12.0–16.0)	<0.001
CKiD (mL/min/1.73 m^2^)	125.3 (116.8–134.1)	94.8 (84.2–105.2)	<0.001
EKFC (mL/min/1.73 m^2^)	112.8 (111.5–114.7)	96.6 (88.2–106.0)	<0.001
CKiD–EKFC (mL/min/1.73 m^2^)	12.1 (4.6–20.1)	−0.9 (−6.6–4.6)	<0.001
Difference between CKiD and EKFC > 95%, N	336 (22.9%)	128 (1.5%)	<0.001
BMI (kg/m^2^)	19.6 (17.3–22.4)	20.3 (18.1–22.9)	<0.001
Systolic BP (mm Hg)	106.0 (99.0–112.0)	107.0 (100.0–114.0)	<0.001
Diastolic BP (mm Hg)	65.0 (59.0–71.0)	67.0 (61.0–72.0)	<0.001
Serum creatinine (mg/dL)	0.50 (0.47–0.56)	0.70 (0.60–0.81)	<0.001
Fasting glucose (mg/dL)	91.0 (87.0–96.0)	90.0 (86.0–950.)	<0.001
HbA1c (%)	5.40 (5.20–5.60)	5.40 (5.20–5.60)	0.883
Total cholesterol (mg/dL)	162.0 (146.0–179.0)	158.0 (142.0–176.0)	<0.001
Triglyceride (mg/dL)	77.0 (55.0–110.0)	74.0 (53.0–103.0)	<0.001

Abbreviations: see Table 1.

## Data Availability

The data presented in this study are available on request from the corresponding authors.
